# Synergizing Ecotoxicology and Microbiome Data Is Key for Developing Global Indicators of Environmental Antimicrobial Resistance

**DOI:** 10.1007/s00248-024-02463-3

**Published:** 2024-11-29

**Authors:** John P. Makumbi, Samuel K. Leareng, Rian E. Pierneef, Thulani P. Makhalanyane

**Affiliations:** 1 Department of Biochemistry, Genetics and Microbiology, University of Pretoria, Pretoria, South Africa; 2 Centre for Epidemic Response and Innovation, School for Data Science and Computational Thinking, Stellenbosch University, Stellenbosch, South Africa; 3 Department of Microbiology, Faculty of Science, Stellenbosch University, Stellenbosch, South Africa

**Keywords:** Antimicrobial resistance, Antimicrobial resistant bacteria (ARB), Antimicrobial resistance genes (ARGs), Bacteria, Microbiomes, Risk assessment

## Abstract

The One Health concept recognises the interconnectedness of humans, plants, animals and the environment. Recent research strongly supports the idea that the environment serves as a significant reservoir for antimicrobial resistance (AMR). However, the complexity of natural environments makes efforts at AMR public health risk assessment difficult. We lack sufficient data on key ecological parameters that influence AMR, as well as the primary proxies necessary for evaluating risks to human health. Developing environmental AMR ‘early warning systems’ requires models with well-defined parameters. This is necessary to support the implementation of clear and targeted interventions. In this review, we provide a comprehensive overview of the current tools used globally for environmental AMR human health risk assessment and the underlying knowledge gaps. We highlight the urgent need for standardised, cost-effective risk assessment frameworks that are adaptable across different environments and regions to enhance comparability and reliability. These frameworks must also account for previously understudied AMR sources, such as horticulture, and emerging threats like climate change. In addition, integrating traditional ecotoxicology with modern ‘omics’ approaches will be essential for developing more comprehensive risk models and informing targeted AMR mitigation strategies.

## Background

**Fig. 1:**
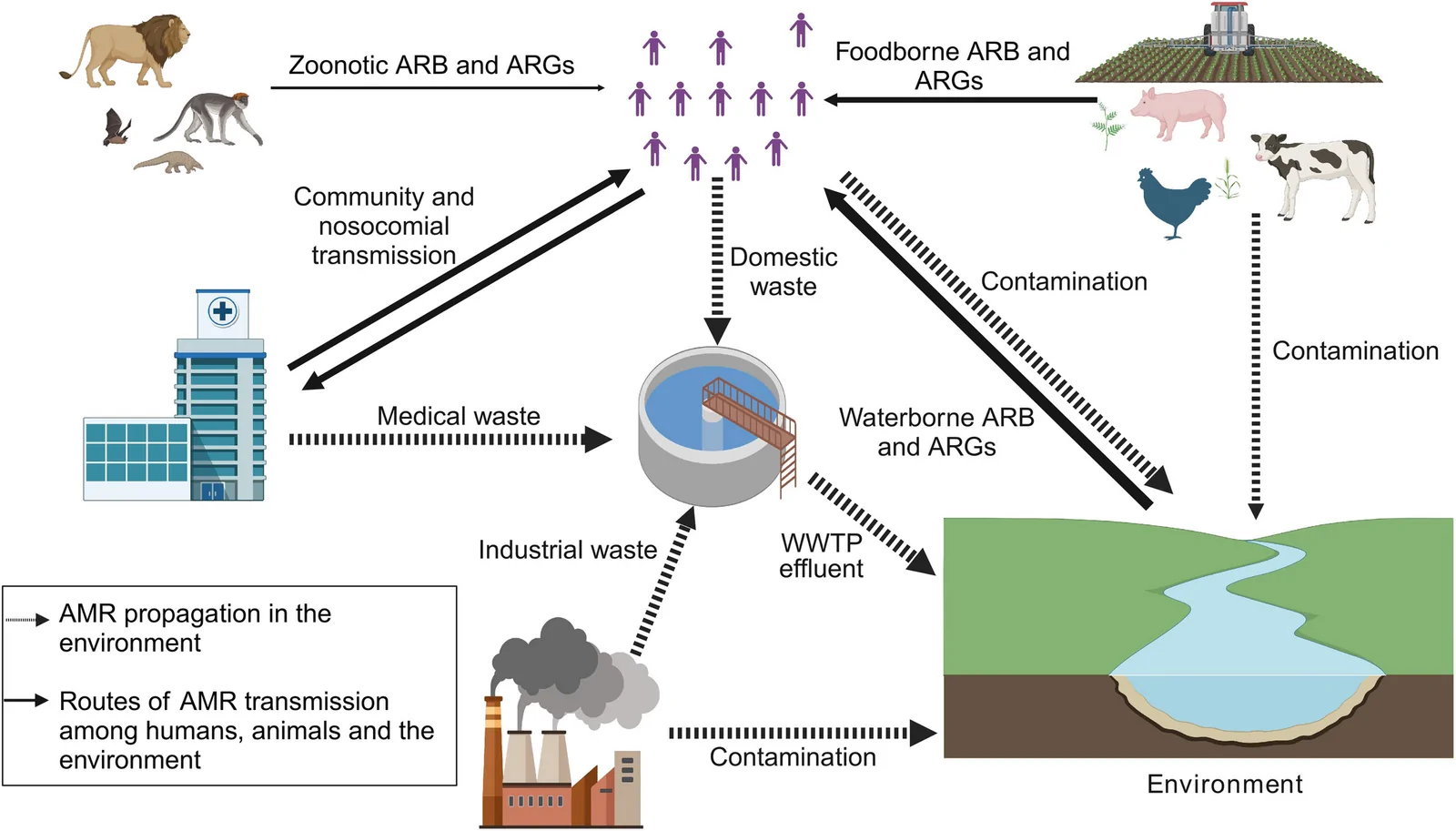
AMR propagation and dissemination pathways due to interconnectivity among humans, animals and the environment. The dashed arrows indicate potential sources of selective pressure, which may drive AMR propagation in the environment, whilst solid arrows represent ARB and ARG transmission routes. The width of the arrows reflects the relative contribution of different sources to AMR propagation and transmission [6, 107–109]. (AMR = antimicrobial resistance; ARB = antimicrobial resistant bacteria; ARGs = antimicrobial resistance genes; WWTP = wastewater treatment plant)

Antimicrobial resistance (AMR) poses a significant threat to public health by compromising the effectiveness of antibiotics and other antimicrobial agents [1]. Several studies have shown a strong correlation between the rise in AMR and increased rates of morbidity and mortality [2–4]. Global estimates indicate that AMR-related deaths could reach 10 million annually by 2050, with economic losses amounting to 3.8% of the global gross domestic product [5, 6], potentially pushing millions into poverty [6, 7]. Additionally, high AMR rates may potentially affect agricultural production in livestock and poultry, impacting food security [8, 9]. The environment, including freshwater sources, agricultural settings like abattoirs and farms, as well as wastewater treatment plants (WWTPs) and landfills, plays a crucial role in the development and dissemination of AMR (Fig. 1) [9–12]. These findings suggest that risk assessments in these environments may be crucial for public health surveillance [13–16].

Despite recognising the importance of environmental factors in AMR transmission, our understanding of the specific ecological drivers behind the spread of antimicrobial resistant bacteria (ARB) and genes (ARGs) remains limited [17, 18]. Current surveillance efforts primarily focus on clinical settings [19], overlooking significant contributions like antimicrobial use in food-producing animals, particularly in regions experiencing rapid agricultural growth like Asia, Africa and South America [20, 21]. The increased antibiotic use in agriculture leads to the release of ARB and ARGs into the environment [22, 23], exacerbating the AMR burden [24–27]. Moreover, the role of horticulture is largely neglected in environmental AMR risk assessments [9, 28–30], despite indications that the use of pesticides and heavy metal-based herbicides may exert selective pressure on plant-associated bacteria, contributing to AMR development [31, 32]. Furthermore, the use of reclaimed wastewater for irrigation and biosolids as fertiliser may lead to contamination of food with ARB [33, 34]. Consequently, agricultural practices that do not directly involve antibiotics may still play a critical role in AMR propagation along the environment-food axis and should be integrated into risk assessments [30].

The increased reports on environmental ARB and ARGs underscores the critical role of environmental surveillance in AMR mitigation [35–37]. Integration of One Health approaches, incorporating agricultural and environmental settings into AMR surveillance is essential [38]. Environmental AMR risk assessment generally involves evaluating the probability of resistance development and the potential consequences of human and animal exposure to ARB and ARGs [16, 38–42]. This may be achieved using mathematical models [43, 44], laboratory experiments [45] and field studies [46]. Although there has been a surge in the number of environmental AMR surveillance studies [47], translating these data into actionable interventions to reduce the AMR burden remains a challenge [16, 48]. This complexity may be attributed to the lack of harmonised and interoperable protocols for AMR risk assessment [47]. Current studies rely on target parameters that are fragmented by lack of capacity, inadequate equipment, limited budgets that affect length of studies, ethics concerns and geography of the sampling sites [49, 50]. These non-standardised protocols may generate highly varied outcomes that are challenging to compare for accurate risk assessment [17, 51].

Urgent action is required to establish common guidelines for environmental AMR surveillance and risk assessment [52, 53]. This will require the harmonisation of risk assessment protocols developed using different methodologies and evaluated in diverse environments and geographical settings. In this review, we explore data concerning global antibiotic usage and AMR drivers, the distribution of environmental ARB and ARGs, as well as the various experimental strategies used to investigate and characterise the human health risk associated with AMR in the environment. Furthermore, we provide a concise overview of the key knowledge gaps and propose parameters that should be considered for the development of standardised AMR surveillance and public health risk assessment tools. We reviewed antimicrobial consumption (AMC) data from the World Health Organization (WHO) and the World Organisation for Animal Health (WOAH) (previously known as the *Office International des Epizooties* or *OIE*) [54, 55] as well as ARG and ARB data (2015–2023) from the National Center for Biotechnology Information’s (NCBI) Pathogen Detection Microbial Browser for Identification of Genetic and Genomic Elements (MicroBIGG-E) (https://www.ncbi.nlm.nih.gov/pathogens/microbigge/#) database. We further explored current strategies for environmental AMR risk assessment by searching two databases: Scopus (search field: article title, abstract, keyword) and PubMed (search field: all fields), for previously published relevant records documenting the use of models or matrices to assess environmental AMR human health risk. The search terms used were (‘antibiotic resistan*’ OR ‘antimicrobial resistan*’) AND (river* OR lake* OR sediment* OR soil OR wastewater OR ‘waste water’ OR ‘river water’ OR WWTP OR ‘wastewater treatment plant’ OR ‘waste water treatment plant’) AND (‘risk assessment’ OR ‘risk characterisation’ OR ‘human health risk’ OR ‘qmra’ OR ‘quantitative microbial risk assessment’). We reviewed studies published between 2015 and the end of February 2024 using the following inclusion criteria: (i) the full text was available in English; (ii) the publication presents primary research; (iii) the research proposes an environmental AMR risk assessment protocol; (iv) the article was published in a peer-reviewed journal (Table 1).

**Table 1:** Previous studies documenting the development of risk assessment tools in environmental AMR research

Location	Methodology	Risk assessment target	Key findings	Reference
China	Proposed a framework for ARG risk ranking in wastewater incorporating average abundance, mobility, host pathogenicity and potential to mediate resistance to antibiotics on the WHO ‘Critically Important Antimicrobials for Human Medicine’ list	ARB and ARGs	The ranking model linked ARGs to priority antibiotics, which suggests the potential role of this framework for use in targeted AMR surveillance	[63]
China	Characterised the environmental risk of ARGs in air, water and soil based on abundance, detection rate and mobility	ARGs	Identified 33 high risk ARGs, with three genes shared across the different environments	[64]
China	Performed source-oriented risk assessment to identify ecological and AMR risk linked to heavy metals	Heavy metals	Anthropogenic sources, particularly industrial and agricultural waste, were the most significant contributors to ecological risks associated with heavy metal contamination	[65]
Ireland	Utilised a risk quotient (RQ) probabilistic method to develop a ranking model for emergence of AMR against macrolide antibiotics at a WWTP	Antibiotic residues	The ranking model allowed the evaluation of an alternative wastewater treatment technology that would reduce the risk of emergence of macrolide resistance at the WWTP	[66]
China	Assessed the seasonal patterns of 12 antibiotics belonging to seven different classes in lake water to guide priority ranking of chemical contaminants	Antibiotic residues	Developed a hierarchical control priority list (HCPL) that can be used to assess the ecological risk of AMR in water environments	[67]
Global	Used metagenomics to identify candidate indicator ARGs for AMR monitoring in wastewater and receiving water bodies	ARGs	Identified minimally redundant ARG targets for AMR monitoring in wastewater-impacted environments	[12]
Ireland	Developed a probabilistic risk ranking model to comparatively assess the predicted amount of antibiotics entering water bodies	Antibiotic residues	Established a protocol to compare the contributions of healthcare and agriculture to antibiotic pollution, and identified highest-ranked antibiotic classes in terms of potential AMR development	[43]
Global	Used metagenomics data and machine learning to calculate ARG risk index based on human accessibility, mobility, pathogenicity and clinical availability	ARGs	Classified ARGs into four categories from highest risk to lowest (Q1, Q2, Q3, Q4) according to likelihood of causing clinical failure	[68]
China	Used metagenomics to determine ARG abundance in treated wastewater and assess ARG removal efficiency by WWTPs	ARGs	Developed an exposure ranking scheme that can be used to assess public health risk of ARGs in WWTP effluent	[69]
France	Investigated source-specific resistance risk of antibiotics in a river basin using positive matrix factorization (PMF) and RQ models	Antibiotic residues	Proposed a reference method for identification and risk ranking of sources of antibiotic pollution	[44]
Taiwan	Developed a computational framework to assess antibiotic resistance risk posed by low-concentration oxytetracycline in aquaculture	Antibiotic residues	The framework allowed the determination of antibiotic concentrations that select for AMR emergence in aquaculture	[70]
China	Performed a risk assessment of ARGs in drinking water systems	ARGs and ARB	Developed a risk assessment matrix taking into consideration chlorine-resistance, ARG transferability and potential pathogenicity of ARG host	[46]
Global	Proposed and evaluated an ‘omics-based’ framework to evaluate ARG risk considering human-associated-enrichment, ARG mobility and host pathogenicity	ARGs, ARB	The risk framework classifies human-associated mobile ARGs as the highest risk with those already present in human pathogens (current threats) categorised as Rank I, ARGs emerging from non-pathogenic bacteria (future threats) classified as Rank II, non-mobile ARGs classified under Rank III, whilst non-human associated ARGs were assigned the lowest risk (Rank IV)	[71]
Brazil	Used a customised reference database to screen for pharmaceutical pollutants from hospital wastewater followed by an in silico quantitative structure–activity relationship (QSAR) model for risk assessment	Antibiotic residues	QSAR model allowed identification of high-risk pharmaceutical contaminants including antibiotics that need to be prioritised for removal from hospital wastewater	[72]
Vietnam	Proposed a probabilistic model to estimate antibiotic resistance development risk (RDR) in aquaculture	Antibiotic residues	The model was able to determine minimum selective concentration risks for each tested antibiotic and classify RDR at the different stages of aquaculture as low, medium or high	[73]
United Kingdom	Used predictive modelling and the class 1 integron as a surrogate marker to determine AMR levels in a river system	ARB, ARGs, MGEs	The model was able to identify areas of the river system with the highest AMR risk thus providing baseline information for prioritisation of mitigation strategies	[10]

## Global Trends in Antibiotic Consumption and Distribution of ARB and ARGs in the Environment

The ‘antibiotic footprint’ concept is widely recognised as a valuable tool for guiding surveillance and risk assessment strategies [20]. Monitoring antibiotic usage is essential for identifying countries and sectors that require antimicrobial stewardship efforts, and for tracking the progress of previous interventions [56]. However, this is constrained by the lack of harmonised global data, as most studies focus on local or regional surveillance [54]. This data gap hampers policy development and the implementation of effective interventions. Additionally, establishing robust surveillance systems presents challenges, particularly in low- and middle-income countries (LMICs), due to resource limitations [49, 54]. Several reports [54, 57] have showed a higher AMR burden in LMICs, despite lower per-person antibiotic consumption compared to high-income countries (HICs). This underlines the need to implement surveillance programmes in these settings.

**Fig. 2:**
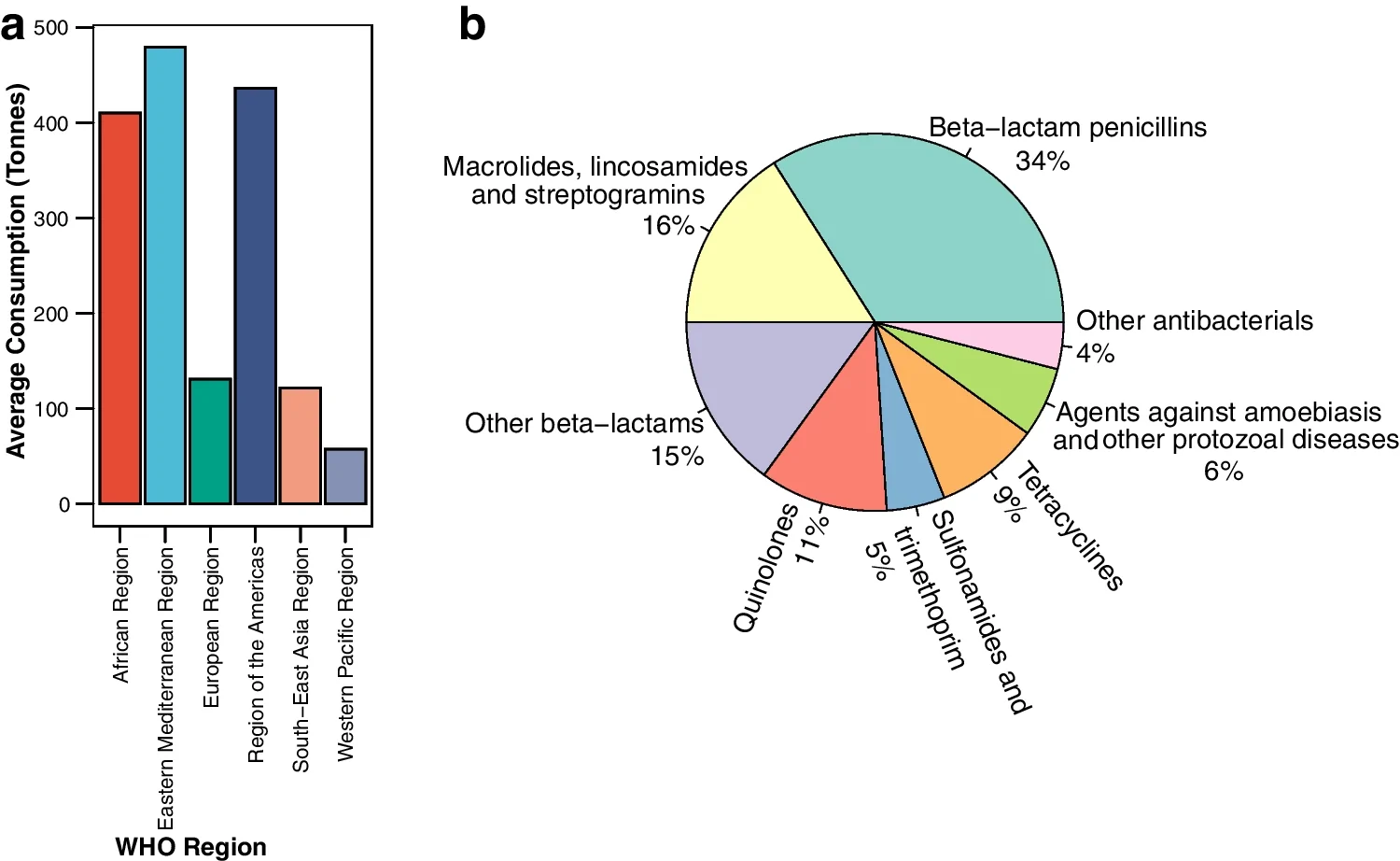
**A** Average consumption of antibiotics for human medicine among WHO regions. **B** Global human antibiotic consumption by class (data sourced from the 2022 WHO GLASS Report [54])

Antimicrobial consumption (AMC) statistics from the World Health Organization (WHO) Global Antimicrobial Resistance and Use Surveillance System (GLASS) and the World Organisation for Animal Health (WOAH) (previously known as the *Office International des Epizooties* or OIE) provide crucial baseline data for monitoring global antibiotic use [54, 55]. Recent data shows that whilst antibiotic usage from HICs has remained stable over the last few years, consumption in LMICs is on the rise [54]. Globally, beta-lactams, macrolides, lincosamides and streptogramins are the most commonly used antibiotics in human medicine (Fig. 2A), whilst tetracyclines are the predominant antibiotics for therapeutic and growth promotion purposes in agriculture [54, 55]. The WHO Eastern Mediterranean Region, the Region of the Americas and the African Region reported the highest average amounts of antibiotic usage (Fig. 2B). However, these data are likely skewed by the very low number of countries, territories and areas (CTAs) (26 out of 193 WHO member CTAs) that submit AMC data. A major knowledge gap exists as participation in these surveillance systems is voluntary, and major world economies such as China and the United States of America (USA) do not currently contribute AMC data. Moreover, many CTAs only report data from public health systems, whilst that from the private sector is still missing [54]. This is significant since the private sector is projected to consume a greater amount of antibiotics than the public sector in many countries [58, 59]. However, despite these limitations, the data available from the existing reports still provides valuable insights into the global antibiotic footprint.

**Fig. 3:**
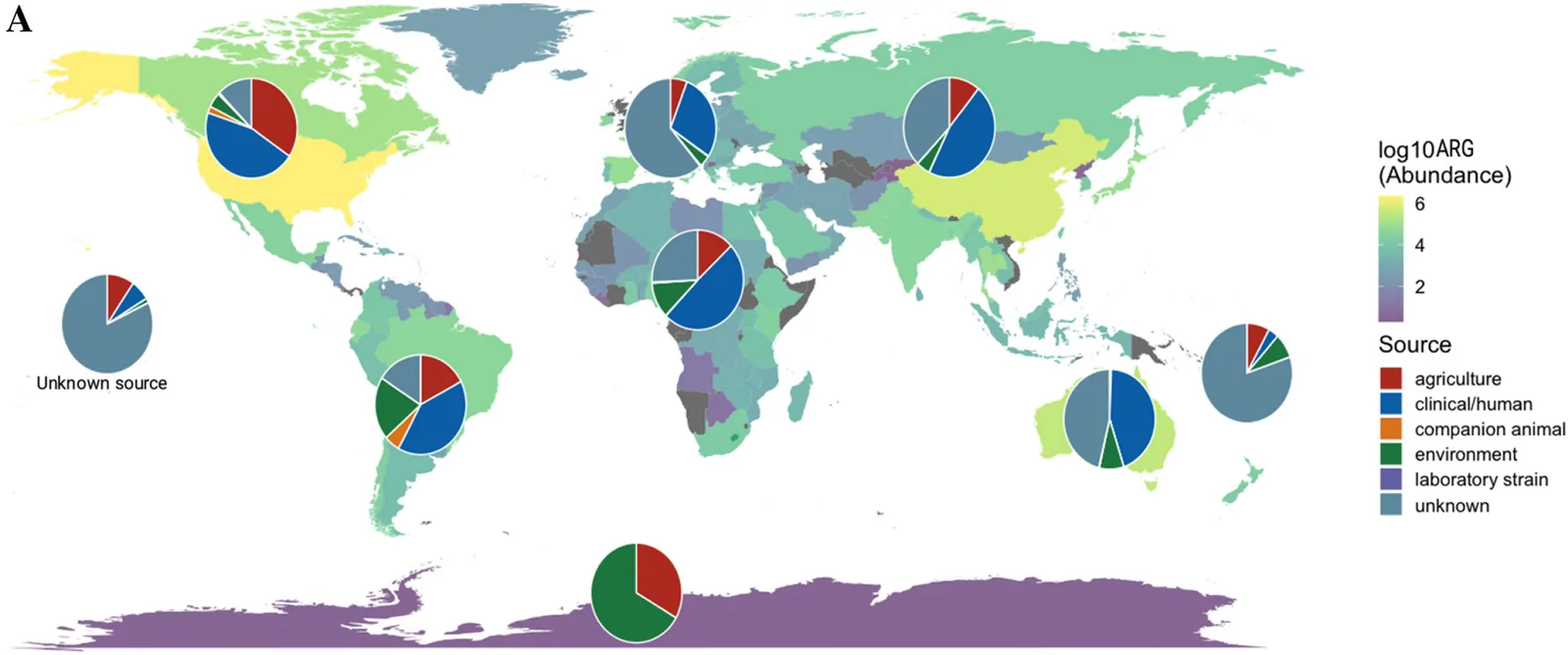

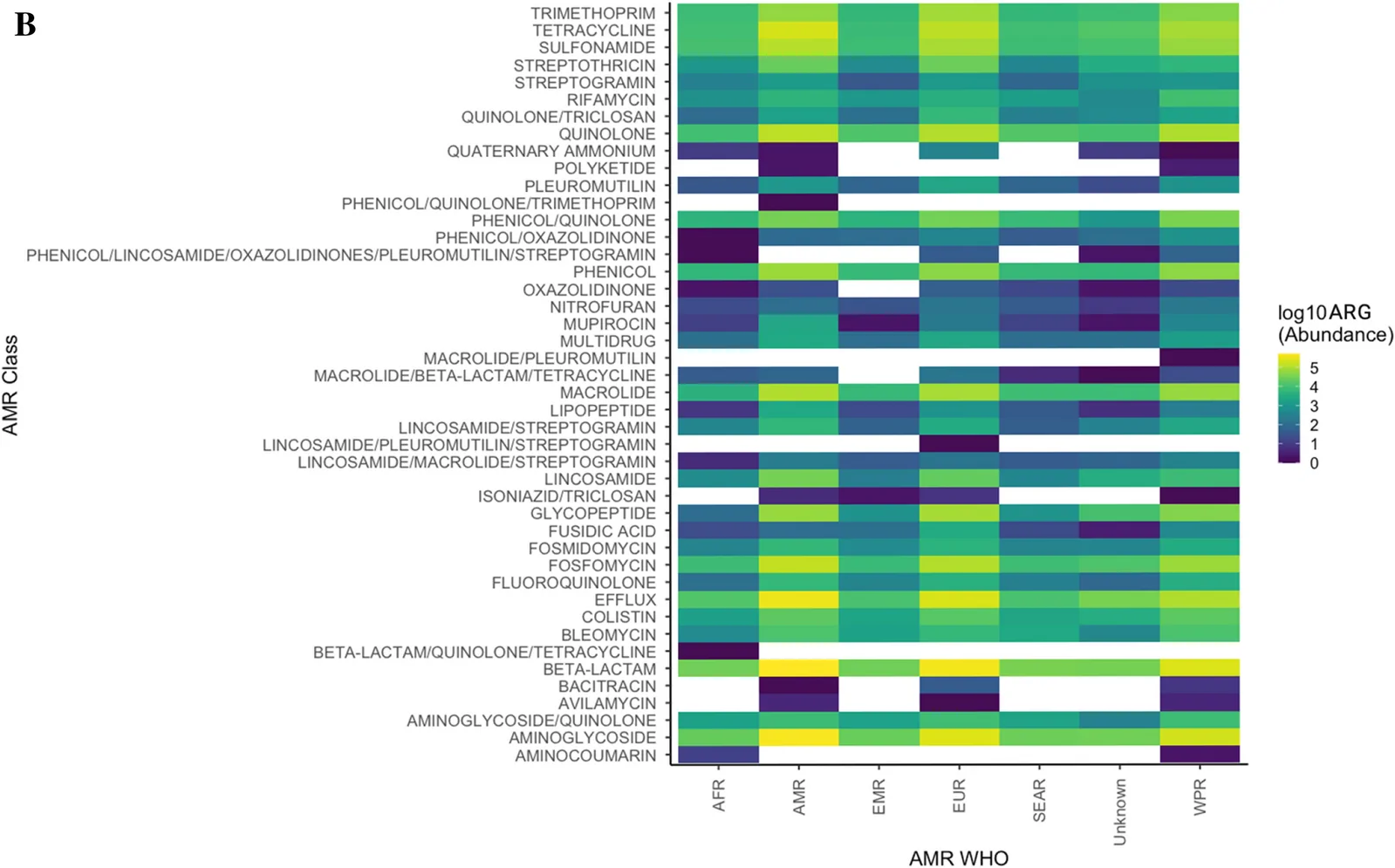
**A** Global data (2015–2023) from NCBI MicroBIGG-E showing the abundance of reported ARB and ARGs across different geographical locations and sources, including agriculture (livestock, poultry and fish), clinical/human (human infections or human-associated commensal bacteria), companion animals, the environment (terrestrial and marine), laboratory strains and undescribed sources. **B** ARGs against human and animal antibiotics reported from the various WHO regions (2015–2023). AFR = African Region, AMR = Region of the Americas, EMR = Eastern Mediterranean region, EUR = European Region, SEAR = Southeast Asian Region, WPR = Western Pacific Region

The number of reported ARGs from the MicroBIGG-E database between 2015 and 2023 showed that high-income WHO regions (European Region, Region of the Americas and Western Pacific Region) showed the most ARGs reported compared to Africa, Eastern Mediterranean and Southeast Asia (Fig. 3A). This confirms that HICs continue to contribute the most to AMR prevalence globally. However, the lower ARG and ARB prevalence reported from LMICs may also be due to less surveillance as a result of resource limitations [60]. Therefore, understanding the true scope of the threat and drivers of AMR in LMICs will require increased investment in public health surveillance programmes. Furthermore, the ARB and ARG patterns correspond to the AMC data above, with genes mediating resistance against aminoglycosides, beta-lactams, tetracyclines, macrolides, lincosamides and streptogramins showing a high prevalence globally (Fig. 3B). This underscores the urgent need to accurately and closely monitor antibiotic consumption to mitigate AMR selection.

**Fig. 4:**
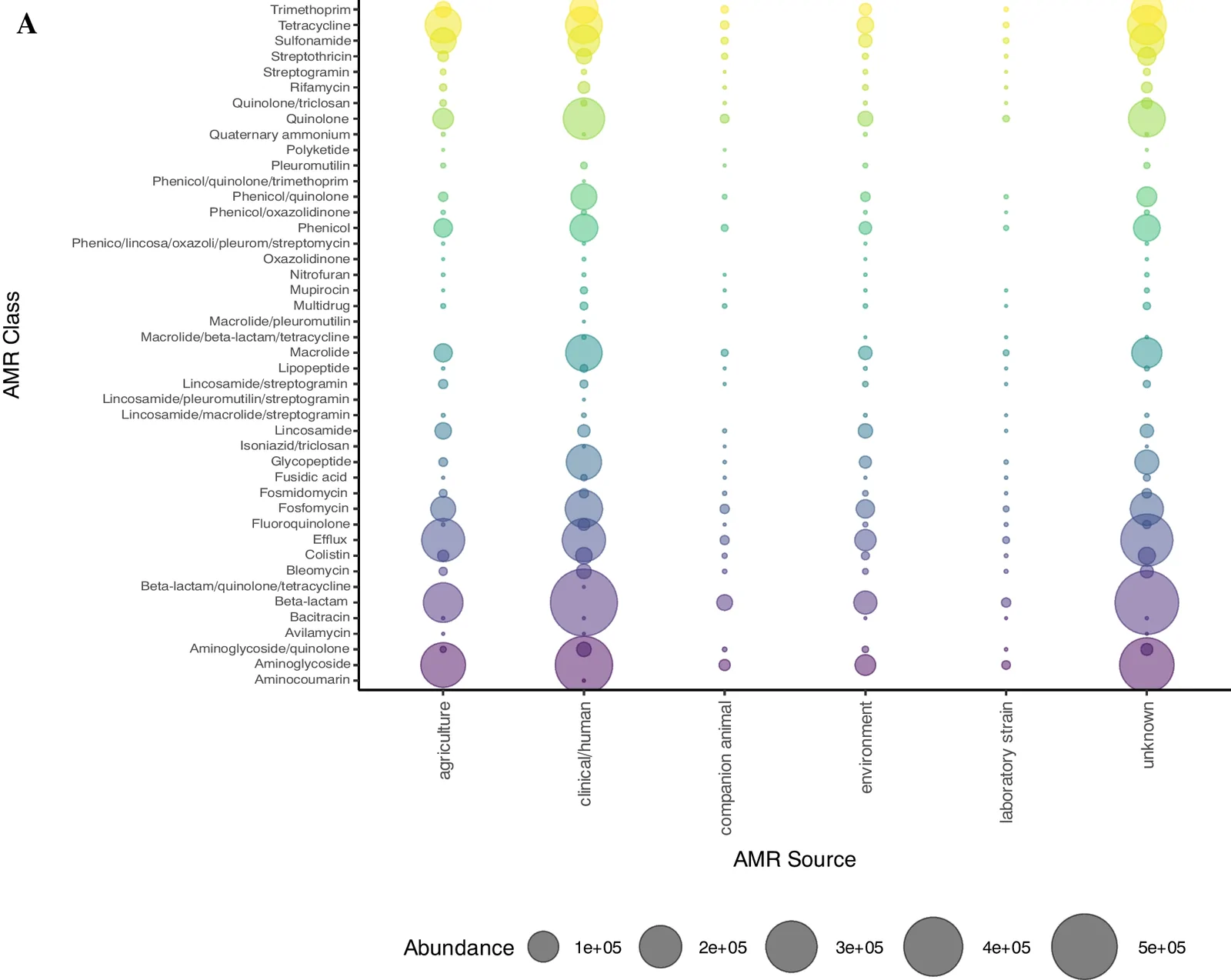

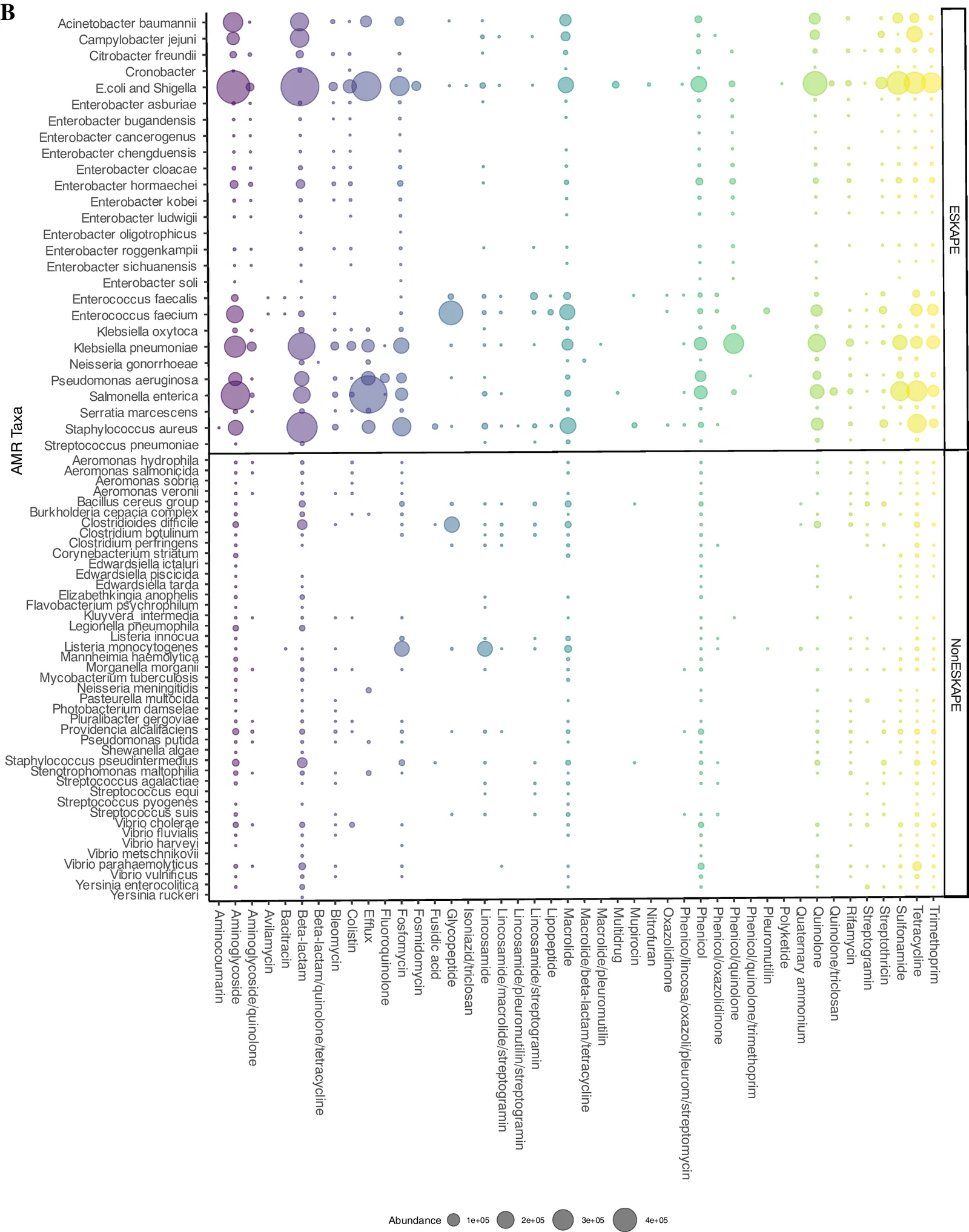
**A** Data from NCBI MicroBIGG-E (2015–2023) showing the contribution of different environments and sources to ARB harbouring genes against human and animal antibiotics (data published between 2015 and 2023). **B** The contribution of ESKAPE and other priority pathogens to the AMR burden. Data sourced from NCBI MicroBIGG-E (2015–2023)

Classification of the ARGs by source showed that although most were reported from clinical and human sources, a considerable proportion of the genes were also from agricultural and environmental sources (Fig. 3A, Fig. 4). Furthermore, the majority of reported ARB are members of the ESKAPE group (*Enterococcus faecium*, *Staphylococcus aureus*, *Klebsiella pneumoniae*, *Acinetobacter baumannii*, *Pseudomonas aeruginosa* and *Enterobacter* sp.) and/or the WHO Priority Pathogens List (PPL) (Fig. 4). These bacteria often show high AMR prevalence, including multidrug resistance [61, 62]. As a result, these pathogens are the leading causes of morbidity and mortality due to AMR. The continued identification of these ARB in agricultural and environmental settings underlines the need to expand risk assessment to include these non-clinical settings.

## Current Strategies for Environmental AMR Risk Assessment and Their Applicability to Public Health

The design and implementation of effective AMR risk assessment requires accurate and timely identification of the sources and drivers of ARGs and ARB [6]. The AMC as well as ARB and ARG prevalence data confirms that non-clinical sources are significant contributors to the AMR burden. This creates a need to develop intervention measures targeted to these settings. Table 1 provides a summary of the various strategies that have been proposed for environmental AMR risk assessment.

Generating reproducible public health data, which allows comparisons across different environments, is crucial for development of efficient AMR risk assessment models [42]. However, as shown in Table 1, a variety of models employing several different techniques and methods have been developed, making standardisation difficult. Importantly, the question of what to monitor remains largely unanswered. There is also a need to harmonise strategies that evaluate the risk of AMR development and those investigating ARB spread and transmission to humans and animals. Nevertheless, ecotoxicology and genomics tools have emerged as the most widely used applications for AMR surveillance and risk assessment [66, 71, 74, 75].

## Evaluating the Risk of AMR Development in the Environment Using Ecotoxicology Techniques

Antibiotic use and the release of chemical pollutants into the environment carry the inherent risk of selecting for AMR in the environment [76]. Currently, many AMR risk assessment methods (Table 1) focus primarily on detecting and quantifying chemical contaminants in the environment. This allows for a quick and cost-effective characterisation of selective pressure, which informs the prediction of AMR development. Chemicals driving AMR in the environment can be divided into three main classes, namely: (i) antibiotics; (ii) heavy metals; and (iii) biocides [40]. Antibiotics are the most targeted chemical pollutants for AMR risk assessment, with fewer studies incorporating detection and quantification of heavy metals and biocides such as disinfectants, quaternary ammonium compounds, pesticides and herbicides, despite their potentially important role in AMR development.

Risk quotient (RQ) models are commonly used for environmental AMR risk assessment [11, 43, 44, 66]. The RQ value is calculated by comparing the predicted or measured environmental concentration (PEC/MEC) to the predicted no-effect concentration (PNEC), and is used to assess the likelihood of chemical contaminants to select for AMR [77]. Risk quotient values ≤ 1 indicate a low likelihood of AMR selection, whilst values ≥ 1 suggest a higher likelihood [66, 78]. By using RQ models, chemicals with the highest potential to select for AMR can be identified, which may guide mitigation strategies [11, 43, 44]. This method can also be used to evaluate the efficiency of pollutant removal, such as at WWTPs [79].

Traditionally, antibiotic minimum inhibitory concentrations (MICs) have been used to determine PNEC values and to predict the likelihood of AMR selection using RQ models [80, 81]. However, selection of resistance may occur at concentrations way below MIC, known as the minimum selective concentration (MSC) [40, 80, 82]. The MSC is defined as the minimum concentration of a chemical required to provide selective advantage to bacteria carrying a resistance gene against the chemical, relative to strains of the same species that do not carry the gene [40]. Minimum selective concentration values may provide a better proxy for the prediction of AMR development due to enhanced sensitivity to low contaminant concentrations [81, 82]. However, more research is needed to determine MSCs for the common AMR-associated chemical pollutants [81]. This is particularly important for heavy metals and biocides, which have received less attention in AMR risk assessment studies compared to antibiotics.

Whilst the methods discussed above are valuable for environmental AMR surveillance, their practical use in risk assessment is limited by the absence of comprehensive, standardised reporting guidelines. Existing frameworks, such as those from the WHO [83] and the United States Environmental Protection Agency (USEPA) [84], offer general guidelines for assessing environmental contaminants. However, these do not specifically address AMR. Instead, they focus on contaminants that can drive selective pressure, indirectly influencing the evolution and spread of ARB and ARGs. Nonetheless, the rising threat of AMR, along with the recognition of ARB and ARGs as emerging contaminants, has prompted the development of more direct guidelines [85, 86]. However, these guidelines are still under development, and their full implementation may take years.

## The Influence of Ecological Factors in Shaping the Environmental Resistome

Environmental factors such as pH, temperature and nutrient availability may influence the rate of acquisition, loss and transfer of ARGs [47]. However, there is little information about the contribution of these factors to AMR, and this remains a largely understudied area [87]. In a recent global study [88], novel indirect positive associations were identified between soil pH and ARG richness, as well as negative correlations between mean annual temperature and soil ARG proportions. Environmental physical and chemical factors may also contribute to AMR propagation through ecological pressure or by affecting the bioavailability and toxicity of pollutants [40]. For example, factors that reduce the bioavailability of antibiotics and heavy metals might result in decreased selective pressure, even when the concentrations of these chemical contaminants are high [40, 89]. Therefore, future studies may need to identify appropriate ecological proxies and incorporate these environmental parameters into AMR risk assessment models.

Furthermore, environmental variations driven by natural processes and human-induced climate change are likely to influence the abundance and expression of AMR systems, including multidrug efflux pumps [40, 90]. These systems play key roles in bacterial adaptation by regulating pH, temperature and quorum sensing in response to environmental changes [40, 88]. This is supported by simulated warming experiments, which demonstrate that rising temperatures can lead to ARG enrichment [91, 92]. Environmental fluctuations may also influence horizontal gene transfer (HGT), potentially increasing or decreasing the spread of ARGs between bacteria [93]. Therefore, incorporating ecological data and climate change models into AMR surveillance can identify key environmental factors that impact ARB/ARG richness, diversity and expression. This can be used to identify proxies for use in risk assessment [88].

## Assessing the Risk of AMR Transmission from the Environment to Humans and Animals

There is some recognition that the possibility of ARB and ARG transmission from the environment, is crucial for developing assessment frameworks (Table 1). However, these exposure assessment studies are complicated by several factors, such as difficulties linked to predicting precise ARB transmission patterns in complex environmental microbiomes [50, 51]. In addition, the high diversity of known ARB and ARGs creates a challenge in the selection of appropriate proxies [94]. Established repositories, such as the Comprehensive Antibiotic Resistance Database (CARD) and ResFinder, have well over 2000 ARGs. These sequences are in addition to several mutations known, or predicted, to mediate AMR [12, 95, 96]. This high number of potential monitoring targets, combined with the unfeasibility of quantifying all targets simultaneously, creates a need to identify candidate ARB and ARGs that may serve as AMR indicators [12]. Moreover, not all ARB and ARGs pose similar or equal threats to public health. Some studies suggest that certain ARGs may be essential in microbial physiology and metabolism [97]. For instance, intrinsic colistin resistance genes, which emerged as a result of chromosomal mutations in lipopolysaccharide synthesis genes in Gram-negative bacteria, demonstrated remarkably low mobility resulting in limited clinical impacts [98, 99]. However, the emergence of plasmid-borne mobile colistin resistance (*mcr*) genes, which are rapidly disseminated via HGT, has limited the use of this critical last-resort antibiotic [98, 99]. Therefore, the mobile ARGs may pose a higher health risk compared with the chromosomal genes.

These substantial challenges may be minimised by focusing primarily on clinically relevant ARB or ARGs associated with severe clinical outcomes, such as those implicated in multidrug resistance [71]. Liang et al. (2020) [100] performed a metagenome-based risk assessment to identify and quantify potentially pathogenic ARB. The authors used intragenomic coexistence pattern analysis to determine the density of metagenome-assembled genomes carrying ARGs, and virulence factor genes in aquatic environments. The drawback to this strategy is that focusing on common ARB may overlook emerging pathogens, limiting the ability to detect and respond to novel outbreaks.

There is increased consensus that the use of indicator ARGs minimises redundancies and enables a more targeted approach [10, 12, 69, 101]. Candidate indicator ARGs can be identified and classified based on several factors, including clinical relevance, gene mobility, association with mobile genetic elements (MGEs), host pathogenicity, geographical ubiquity and risk of human exposure [10, 68, 71]. Zhang et al. (2021) [71] proposed a framework in which mobile ARGs mediating multidrug resistance, and carried by human-associated pathogenic bacteria, rank as posing the greatest risk. Non-mobile ARGs, and those carried by non-human associated bacteria, rank low-risk under this framework. Similarly, Zhang et al. (2022) [68] suggested a risk index that considers antibiotic use, the range of antibiotics to which an ARG mediates resistance, gene mobility, ARB pathogenicity and human accessibility. According to this approach, multidrug resistance genes pose the highest risk, whereas ARGs carried by non-pathogens pose the lowest. These frameworks may contribute significantly to advances in risk assessment as they are suitable for both qPCR and metagenomic datasets, and can also be applied in low-resource settings.

Mobile genetic elements such as plasmids, insertion sequences, pathogenicity islands and bacteriophages are prevalent in the environment and facilitate ARG transfer between bacteria [102, 103]. Delgado-Baquerizo et al. (2022) found that MGEs were the most important factor associated with soil ARG proportion, relative to other environmental factors such as climate and location. Therefore, MGEs may provide an ideal proxy for ARG mobility and prevalence. However, many studies focus cell-associated MGEs, whilst not taking into account those carried on free-floating extracellular DNA (exDNA) [102, 104]. Several studies have identified a higher proportion of ARGs carried on exDNA than cell-associated DNA in the environment, underlining the importance of MGEs in AMR propagation and transmission [104–106]. Processes such as wastewater treatment and changes in temperature and pH may cause cell lysis, leading to the release of ARGs carried on MGEs [102, 104]. These ARGs can be subsequently taken up by competent cells under favourable conditions, further leading to AMR propagation [105]. Therefore, the role of exDNA should be considered when evaluating the transmission potential environmental ARGs.

## Summary

Here, we review current insights on human health risk assessment strategies of environmental resistomes. These strategies aim to identify and mitigate sources of AMR emergence and transmission. However, our synthesis of current studies suggest that the effectiveness of these approaches is variable in terms of their public health utility and applicability. The lack of standardised surveillance and risk assessment strategies presents a difficulty when attempting to draw comparisons among different environments, countries and regions. In addition, the lack of available data from HICs, that are predicted to use high amounts of antibiotics annually, hinders country-country comparisons. Concurrently, poor surveillance in LMICs may obscure the precise threats of AMR in these countries. The risk assessment strategies discussed were developed and evaluated in HICs, and the applicability in LMICs remains unclear. Given the increased risks of AMR in LMICs, it is crucial to develop appropriate risk assessment tools tailored to local needs and resources.

Furthermore, the risk characterisation strategies presented here were primarily developed and validated in aquatic environments. Therefore, the efficacy of these strategies in other environments remains unclear. To investigate the risk of AMR in the environment, it is essential to identify appropriate benchmarks or proxies. These proxies may include MIC, MSC, the abundance of ARB/ARGs and environmental factors such as temperature and pH. However, these approaches may cross disciplinary methodological boundaries and include diverse proxies for determining risks to human health. Therefore, it is vital that these approaches are standardised to enable improved reproducibility and comparability across different environments and spatial–temporal scales. Additionally, AMR-specific ecotoxicological guidelines must be developed, accounting for chemical drivers of resistance, as well as other sources like horticulture and factors like climate change.

The integrated use of traditional ecotoxicology techniques and modern ‘omics’ tools may provide a more holistic understanding of the risks linked to AMR emergence and spread. We propose that assessment studies should firstly determine the baselines linked to AMR selection, followed by evaluating the probability of transmission to at-risk populations. Risk assessment models must also provide well-defined, and actionable, interventions that maximise resource prioritisation. The success of such approaches will rely on continuous monitoring to identify novel proxies and to evaluate the effectiveness of previously implemented interventions.

## Data Availability

The data used in this article are publicly available and have been cited in the relevant sections of the manuscript.
